# Integrated multi-omics, spatial profiling and organoid modeling drive transformative advances in chronic liver disease and hepatocellular carcinoma immunomicroenvironment research

**DOI:** 10.3389/fimmu.2026.1743439

**Published:** 2026-01-26

**Authors:** Qi Liu, Chenyu Wang, Cheng Ye, Huawei Zhang, Teng Li, Wenjuan Wei, Senyan Wang, Huapeng Zhang

**Affiliations:** 1 Translational Medicine Centre, The First Affiliated Hospital of Zhengzhou University, Zhengzhou, China; 2 Department of Hepatobiliary and Pancreatic Surgery, The First Affiliated Hospital of Zhengzhou University, Zhengzhou, Henan, China; 3 The First Affiliated Hospital of Zhengzhou University, Zhengzhou University, Zhengzhou, Henan, China; 4 Department of Neurosurgery, Beijing Chao-Yang Hospital, Capital Medical University, Beijing, China; 5 Department of Ophthalmology, The First Affiliated Hospital of Zhengzhou University, Zhengzhou, China; 6 Department of Hepatobiliary and Pancreatic Surgery, The First Affiliated Hospital of Zhengzhou University, Zhengzhou, Henan, China

**Keywords:** CLD(Chronic liver disease), hepatocellular carcinoma, immune microenvironment, liver organoids, single-cell sequencing, spatial transcriptomics

## Abstract

Chronic liver disease (CLD) represents a major global public health challenge, necessitating a systematic understanding of its complex immunopathological mechanisms. This review comprehensively summarizes the groundbreaking applications of cutting-edge technologies—including single-cell sequencing, spatial transcriptomics, and organoid models—in chronic liver disease immunology research: Single-cell sequencing resolves immune cell heterogeneity at unprecedented resolution, identifies rare cell subsets, and reveals dynamic changes and regulatory networks through multi-omics integration; Spatial transcriptomics complements this by mapping immune-stromal interactions within structural contexts such as the portal tract, fibrotic septa, and tumor niches, uncovering spatially organized immune evasion mechanisms and microenvironmental remodeling; Organoid technology constructs humanized liver-immune models that recapitulate disease-specific features—such as fibrosis, steatohepatitis, and hepatocellular carcinoma—enabling mechanistic validation, drug screening, and individualized therapeutic exploration. The synergistic integration of multi-omics profiling, spatial mapping, and organoid modeling is driving a paradigm shift in chronic liver disease immunology—transitioning from static cellular descriptions to spatiotemporal mechanism decoding, and from population-level insights to individualized pathophysiology and treatment prediction. These advanced approaches establish a technological foundation for building precision immunotherapeutic strategies tailored to spatiotemporal regulation of the liver immune microenvironment.

## 1 Introduction

Chronic liver diseases(CLDs), including viral hepatitis, metabolic dysfunction-associated steatotic liver disease(MASLD), and autoimmune liver diseases, collectively represent a major global health burden (1). Their progressive nature often leads to liver fibrosis, cirrhosis, and hepatocellular carcinoma(HCC). Central to this pathogenesis is the persistent dysregulation and remodeling of the hepatic immune microenvironment—a highly heterogeneous and dynamic ecosystem (2). Conventional research tools have offered limited capacity to systematically decipher its cellular composition, spatial architecture, and molecular network interactions, which has hindered a deeper understanding of disease mechanisms and the development of targeted therapies.

Recent advances in technologies provide new opportunities to resolve this complexity. Single-cell sequencing(scRNA-seq) now enables high-resolution mapping of hepatic cellular landscapes, allowing identification of rare pathogenic cell subsets and their dynamic trajectories (3). Spatial transcriptomics complements this by preserving tissue context, permitting *in situ* analysis of immune cell distribution and interactions within anatomical niches such as lobules, portal tracts, and fibrous septa (4). Meanwhile, organoid models recapitulate human liver structure and pathology ex vivo, offering a versatile platform for mechanistic investigation and personalized drug testing under controlled conditions (5).

**Figure 1:**
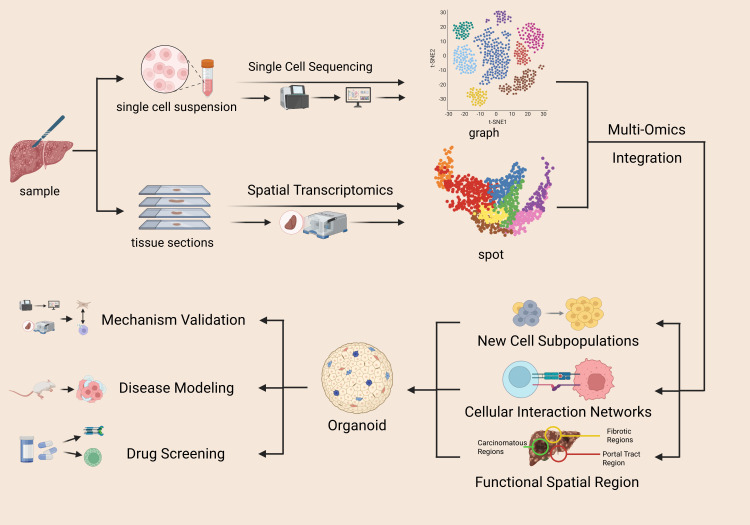
Technical workflow and core analysis dimensions for immune microenvironment research in CLDs. This Figure outlines the core technical system and analytical framework for studying the immune microenvironment in CLDs. ScRNA-seq acquires single-cell suspension data, identifies new cell subpopulations via t-SNE clustering, and analyzes cellular heterogeneity through multi-omics integration. Spatial transcriptomics uses tissue section spot sequencing to map cell distribution and interaction networks in functional regions (portal tracts, fibrotic regions, carcinomatous foci). Organoid technology enables disease modeling, mechanism validation, and drug screening. These three technologies synergistically form a complete research chain from cell resolution to spatial localization and functional validation.

While each of these approaches has generated valuable insights independently, the full complexity of the liver immune microenvironment cannot be fully captured by any single method (6). A key challenge—and opportunity—for the field is the integrative analysis of multi-dimensional, multi-scale data. Synthesizing information across these layers is essential to build a coherent model that connects molecular events, cellular behaviors, tissue organization, and organ function (7). This review aims to systematically summarize key discoveries enabled by scRNA-seq, spatial transcriptomics, and organoid technology in the study of CLD immunology. It also discusses future directions for combining these technologies to elucidate regulatory mechanisms, identify novel therapeutic targets, and ultimately advance toward personalized immunotherapy. Together, these approaches form an integrative pipeline from cellular dissection to spatial mapping and functional validation (**Figure 1**).

## 2 ScRNA-seq

ScRNA-seq enables the systematic analysis of cellular heterogeneity, dynamic changes, and molecular regulatory networks within the immune microenvironment of CLDs at an unprecedented single-cell resolution. It has become a core technology driving a paradigm shift in this research field. Its technical advantages lie not only in the precise identification of rare immune cell subsets undetectable by traditional bulk sequencing—such as CD39+ double-negative T (DNT) cells and Th1-like cells—but, more importantly, in its ability to reveal continuous cell state transition trajectories and regulatory mechanisms by integrating multi-omics data like transcriptomics, epigenomics, and proteomics. These capabilities elevate scRNA-seq from merely describing cell types to dynamically dissecting the spatiotemporal remodeling mechanisms of the immune microenvironment, providing a solid data foundation for precise immune subtyping and targeted intervention strategies in CLDs. Beyond single-modal scRNA-seq, multimodal single-cell approaches—such as CITE-seq, scATAC-seq, and SHARE-seq—have revolutionized the characterization of hepatic immune cells. These technologies enable simultaneous capture of multiple molecular layers, revealing how epigenetic states and protein expression profiles synergistically define cell identity and functional plasticity in CLDs. For example, CITE-seq has refined the classification of liver macrophages by integrating transcriptomic signatures with surface marker expression, resolving previously ambiguous subpopulations like LPAMs and SAMs (8).

### 2.1 B cells and autoantibodies

High-titer autoantibodies are a hallmark of autoimmune liver diseases. Specifically, antinuclear antibodies (ANA) and anti-mitochondrial antibodies (AMA) are commonly found in primary biliary cholangitis (PBC), where they can directly attack bile duct epithelial cells, leading to chronic cholestasis and fibrosis (9). Anti-liver-kidney microsomal type 1 antibodies (anti-LKM1) are associated with autoimmune hepatitis (AIH), and their presence suggests T cell and B cell-mediated hepatocyte damage. ScRNA-seq is being applied to directly dissect the pathological role of B cells in autoimmune liver disease. For example, a study integrating multi omics methods performed scRNA-seq on immune cells of AIH patients, which not only confirmed the overall expansion of B cell population in the peripheral blood of patients, but more importantly, identified a specific AIF1+ B cell subset that was significantly enriched in AIH. This study directly correlated specific B cell subsets (AIF1+ B cells) with the disease status of AIH through single-cell resolution data, providing a new entry point for understanding the cellular immune abnormalities behind the production of autoantibodies. This demonstrates the powerful ability of scRNA-seq in revealing the complex immune microenvironment of the autoimmune liver, especially the heterogeneity of B cells (10).

In patients with chronic viral hepatitis, such as hepatitis C, the detection rate of autoantibodies is significantly elevated and correlates with disease activity and treatment response (11). Studies indicate that approximately 20-30% of patients with chronic hepatitis C (CHC) have detectable ANA or anti-LKM1 in their serum, suggesting these antibodies may be directly induced by HCV triggering an autoimmune response, rather than being solely caused by interferon therapy (12). Similarly, chronic HBV infection can lead to liver fibrosis and cirrhosis, during which autoantibodies produced by B cells may exacerbate liver injury and disease progression.

In-depth investigation of B cell function reveals that in chronic hepatitis B (CHB) or CHC, CD19^+^ memory B cells, as key players in hepatic immune memory, are enriched within the liver and participate directly in viral clearance by secreting antiviral antibodies (e.g., anti-HBV or anti-HCV antibodies)(**Table 1**). It is important to note that the long-term survival of these cells depends on specific signals in the liver microenvironment such as BAFF and TACI, and their functional state is closely related to the efficiency of the patient’s immune response (13). Studies combining scRNA-seq and B cell receptor (BCR) sequencing have found that clonal expansion of CD19^+^ memory B cells is directly linked to dynamic changes in antibody levels in patients with chronic hepatitis (14). Furthermore, dendritic cells (DCs), acting as a critical bridge between innate and adaptive immunity, also play a central role in CLDs. In chronic hepatitis, CD11c^+^ DCs recruit and activate T cells and B cells by expressing the chemokine CXCL13 and costimulatory molecules like CD80/CD86, thereby guiding B cells to migrate into tertiary lymphoid structures (TLS) to promote the production of antiviral antibodies. This mechanism is crucial for maintaining liver immune homeostasis (15). TLS are transient immune cell aggregates formed in non-lymphoid tissues, participating in local immune responses. They are composed of B cells, T cells, dendritic cells, and stromal cells, functioning similarly to lymph nodes, and are commonly found in chronic inflammation, infection, or tumor microenvironments.

**Table 1:** Key immune cell subpopulations in CLDs: characteristic features, major functions, and associated diseases.

Cell type	Characteristic subset	Main functions	Associated diseases
B cells	CD19^+^ memory B cells	Secreting antiviral antibodies, maintaining immune memory	CHB, CHC
T cells	Th1-like cells	Pro-inflammatory, damaging bile duct epithelial cells	PBC
	IFNγ^+^ CD8^+^ T cells	Antiviral effector function, prone to exhaustion	Viral Hepatitis
	CXCL13^+^ T cells	Recruiting B cells, promoting germinal center formation	CHB-related Fibrosis
	CXCR6^+^ NKT cells	Early: Anti-fibrotic Late: Pro-fibrotic	Liver Fibrosis
	FOXP3^+^ Tregs	Functional dysregulation/Immunosuppression	PBC, HCC
Macrophages	M1-type	Pro-inflammatory, Pro-fibrotic	Various CLDs
	M2-type/CD206^+^/TREM2^+^ CD9^+^ SAMs	Pro-fibrotic	MASLD, Fibrosis, MASH
	CX3CR1^+^ CD63^+^ LPAMs	Anti-inflammatory, Neuroprotective	MASH
Other	Neutrophils (NETs)	Direct liver injury	Alcoholic Hepatitis

This table summarizes core immune cell subpopulations in CLDs, their key traits, functions and linked conditions: CD19^+^ memory B cells (secrete antiviral antibodies, maintain immune memory; CHB, CHC); T cell subsets including pro-inflammatory, bile duct-injuring Th1-like cells (PBC), antiviral but exhaustion-prone IFNγ^+^CD8^+^ T cells (viral hepatitis), B cell-recruiting, germinal center-promoting CXCL13^+^ T cells (CHB fibrosis), early anti-fibrotic/late pro-fibrotic CXCR6^+^ NKT cells (liver fibrosis), and dysfunctional/immunosuppressive FOXP3^+^ Tregs (PBC, HCC); macrophages like pro-inflammatory/pro-fibrotic M1-type (multiple liver diseases), pro-fibrotic M2/CD206^+^ type (MASLD, fibrosis), anti-inflammatory/neuroprotective CX3CR1^+^CD63^+^ LPAMs (MASH), pro-fibrotic TREM2^+^CD9^+^ SAMs (fibrosis, MASH); and neutrophils (form NETs, cause direct liver injury; alcoholic hepatitis).

### 2.2 T cell subsets and functional abnormalities

Analysis of T cell populations shows that in patients with PBC, the CD4^+^ T cell subset shifts, with the expansion of pro-inflammatory Th1-like cells closely associated with bile duct epithelial cell injury(**Table 1**). These Th1-like cells are primarily confined to the liver tissue, and their numbers positively correlate with disease severity. In fact, multiple T cell subsets exhibit enhanced inflammatory responses, particularly pronounced in Th1-like cells, indicating their major pro-inflammatory role in PBC pathogenesis. Notably, although the number of regulatory T cells (Tregs) increases in PBC patients, their functional genes display a paradoxical activation state, involving these cells in both immunosuppressive and immune-activating processes. This results in Tregs being unable to effectively perform their traditional immunosuppressive functions, suggesting that Treg dysfunction may be a key factor in the breakdown of immune tolerance (9). Additionally, liver sinusoidal endothelial cells (LSECs), upon stimulation by liver injury, upregulate MHC-II molecules and present antigens to CD4+ T cells. This process indicates that LSECs possess antigen-presenting capabilities and can activate autoreactive T cells, thereby promoting disease progression (16). On the other hand, in HCC, infiltrating γδ T cells exhibit LAG3-dependent dysfunction. Overexpression of LAG3 may contribute to immunometabolic dysregulation by suppressing the metabolic phenotype of effector T cells (17).

In recent years, scRNA-seq has provided a high-resolution perspective for analyzing the function and mechanisms of specific T cell subsets in CLDs, revealing the central roles of various gene-positive T cell populations at different disease stages. In chronic viral hepatitis (e.g., CHB or CHC), IFNγ-positive CD8+ T cells are the core effector cells of antiviral immunity (**Table 1**). They directly kill infected hepatocytes by secreting IFNγ and induce the activation of type I interferon signaling pathways (e.g., ISG genes). However, persistent viral infection leads some IFNγ+ CD8+ T cells into a functionally exhausted state (e.g., PD-1+ Tim-3+), resulting in significantly reduced secretory capacity, thereby promoting viral persistence and liver injury (18). Combining scRNA-seq with T cell receptor (TCR) sequencing has confirmed that their clonal expansion is directly associated with the response rate to antiviral therapy, providing molecular markers for monitoring treatment efficacy (19). Furthermore, RNA velocity and lineage tracing tools have become indispensable for dissecting the dynamic differentiation trajectories of hepatic immune cells. By modeling the splicing dynamics of pre-mRNA and mature mRNA, RNA velocity predicts the future functional state of cells, revealing the transition of naive T cells to exhausted T cells in chronic viral hepatitis or the differentiation of monocytes into pro-fibrotic SAMs during liver fibrosis. Combined with TCR/BCR clonal tracing, these tools have identified clonally related T cell subsets that transition between anti-inflammatory and pro-inflammatory states across disease stages, providing a dynamic perspective on immune cell plasticity in CLDs. For instance, lineage tracing has confirmed that CXCR6+ NKT cells originate from a common precursor pool and switch their functional phenotype in response to persistent liver injury, validating their dual role in fibrosis progression (20). Similarly noteworthy, in the progression of liver fibrosis in CHB patients, CXCL13-positive T cells such as T follicular helper (Tfh) cells and memory T cells guide T cell migration into the liver via secretion of CXCL13 and expression of its receptor CXCR5 (**Table 1**). These cells produce high levels of HBV-specific interferon (IFN)-γ and IL-21, possess stronger antiviral capacity, promote the formation of local immune memory and the durability of antiviral immunity, and may indirectly influence the production of anti-fibrotic antibodies by recruiting B cells to form germinal centers (21).Overall, these findings not only deepen the understanding of immune mechanisms in CLDs but also provide a theoretical basis for immunotherapy strategies targeting specific T cell subsets (e.g., PD-L1+ TAMs, IL-17A+ γδ T cells, IFNγ+ CD8+ T cells, CXCL13+ T cells, and CXCR6+ NKT cells). Furthermore, research has identified that CD4^+^ FOXP3^+^ T cells, a core Treg subset, play a pro-tumor role by suppressing the immune microenvironment in HCC progression (**Table 1**): these cells are specifically enriched in the carcinomatous areas of the liver, where they impair anti-tumor immune responses through their classic immunosuppressive functions. They interact with PD-L1-expressing LAMP3^+^ dendritic cells and are selectively retained by the tumor microenvironment, collectively maintaining an immunosuppressive environment favorable for tumor growth (22).

### 2.3 Macrophages and neutrophils

During the progression of CLDs, immune cell dysfunction is closely linked to the driving mechanisms of liver injury and fibrosis. Macrophages, as central immunoregulatory cells, exhibit a dual role through abnormal polarization states: on one hand, under hepatocyte activation conditions, macrophages can polarize towards the pro-inflammatory M1 phenotype, directly exacerbating liver inflammation and promoting fibrosis via upregulation of signaling pathways like YAP/TAZ/CYR61 (**Table 1**); on the other hand, during the transition from metabolic dysfunction-associated steatotic liver disease (MASLD) to cirrhosis, macrophages can polarize towards the M2 phenotype, accelerating liver fibrosis and cirrhosis by secreting prostaglandin E2 (PGE2), which binds to the EP4 receptor, further activating HSC autophagy and proliferation (16) (**Table 1**). In summary, this dynamic imbalance in M1/M2 polarization not only reflects the contradictory role of macrophages in liver disease but also reveals their complex regulation of the liver microenvironment across different pathological stages.

The in-depth application of scRNA-seq has further refined our understanding of macrophage heterogeneity. Recent studies using scRNA-seq in CLDs have revealed the critical roles of several specific gene-positive macrophage subpopulations, providing new perspectives on the complexity of the liver immune microenvironment. Firstly, in metabolic dysfunction-associated steatohepatitis (MASH), CX3CR1^+^CD63^+^ liver portal-associated macrophages (LPAMs) have garnered attention for their unique anti-inflammatory and neuroprotective functions (**Table 1**). These macrophages delay MASH inflammatory progression by inhibiting neutrophil infiltration; simultaneously, they closely interact with hepatic sympathetic nerve endings, maintaining the structural integrity of sympathetic nerves and providing neural support for liver immune homeostasis. Their loss leads to increased inflammatory granulocyte infiltration and exacerbated sympathetic neurodegeneration (23). Secondly, in the fibrotic stage, CD206^+^ macrophages display functional duality: they maintain local immune homeostasis by phagocytosing apoptotic cells and debris, yet they are significantly enriched in fibrotic areas, express the typical M2 macrophage marker CD206, and drive HSC activation and collagen deposition by secreting factors like Thbs1, which binds to integrin receptors on HSCs, highlighting their potential as interventional targets (24) (**Table 1**). Furthermore, scar-associated macrophages (SAMs) are a monocyte-derived population expressing TREM2/CD9 that specifically expands in fibrotic regions (**Table 1**). They directly activate HSCs by secreting factors like SPP1 and PDGFB, promote collagen deposition, and form interaction networks with other cells within the scar, synergistically driving fibrosis progression. This conserved cell population offers a new target for anti-fibrotic therapy (7). Collectively, these studies indicate that macrophage subsets with different genetic markers have distinct functional division at various stages of CLD (e.g., MASH, fibrosis, cirrhosis). They may delay disease progression through anti-inflammatory or neuroprotective actions, or exacerbate pathological damage due to pro-fibrotic effects or dysregulated immunomodulation, providing important rationale for developing targeted macrophage therapies.

Meanwhile, abnormal activation of other immune cells should not be overlooked. For instance, in alcoholic hepatitis, neutrophil infiltration and their ability to form neutrophil extracellular traps (NETs) have been clearly associated with the degree of liver injury (**Table 1**). Further analysis shows this heterogeneity is manifested as high-density and low-density neutrophils possessing highly activated or exhausted transcriptomic signatures, respectively, further highlighting the dysregulated crosstalk among multiple immune cell types within the CLD immune microenvironment (25). Therefore, whether it’s the skewed polarization of macrophages or the aberrant activation of neutrophil functions, the dysregulation of these immune cells collectively contributes to the persistent worsening of liver fibrosis and drives cirrhosis towards irreversible stages.

## 3 Spatial transcriptomics

### 3.1 Technical advantages

While scRNA-seq reveals cellular heterogeneity, understanding the spatial distribution and interactions of these cells within their native tissue context is equally crucial. Spatial transcriptomics has emerged to directly address the lack of spatial context in single-cell technologies by capturing transcriptome-wide data within preserved tissue architecture (26). By integrating whole transcriptome data with spatial location information, spatial transcriptomics overcomes the spatial context limitation of traditional sequencing methods, enabling *in situ* analysis of the liver immune microenvironment in two or even three dimensions. This approach has proven particularly powerful in CLDs, where it can delineate disease-specific transcriptional landscapes within anatomical structures such as fibrotic septa (27). Consequently, this technology can not only precisely map the distribution patterns of immune cells in structural regions like liver lobules, portal tracts, and fibrous septa but also reveal region-specific immune-stromal interaction networks. For example, by analyzing the spatial proximity and ligand-receptor pair expression between macrophages and activated HSCs at the fibrotic front, it can elucidate the local transmission mechanisms of pro-fibrotic signals. Indeed, integrative single-cell and spatial analyses have been indispensable for defining the cellular niche that drives fibrosis in human cirrhosis (7). Simultaneously, the technology can identify the coupling between cell states and spatial locations within micro-regions like germinal centers or inflammatory foci, providing an indispensable technical perspective for understanding the regionalized regulation and structural remodeling of immune responses in CLDs as demonstrated in the spatial mapping of immune-stromal interfaces in primary sclerosing cholangitis (28). In recent years, the advent of high-resolution spatial transcriptomics platforms like Visium HD and Xenium has achieved subcellular resolution, allowing clear distinction of transcriptomic features from LSECs, hepatocytes, and infiltrating immune cells. Furthermore, multi-omics integration technologies like DBiT-seq can simultaneously obtain transcriptome and proteome information from the same tissue section, providing a powerful tool for spatially resolved molecular phenotyping of liver immune cells (29). Notably, the rapidly evolving multiplexed spatial platforms—including MERFISH, seqFISH+, and CosMx SMl—have further pushed the boundaries of spatial resolution and gene detection throughput beyond conventional spatial transcriptomics. These platforms enable quantitative mapping of thousands of target genes at subcellular resolution, allowing precise delineation of cell-cell communication networks within heterogeneous liver microenvironments that were previously undetectable. Complementing these transcriptomic advances, spatial epigenomics technologies such as CUT & Tag-spatial and ATAC-spatial integrate spatial localization with epigenetic signatures, uncovering region-specific chromatin accessibility and histone modification patterns in key cell types. This integration bridges the gap between spatial gene expression and its regulatory mechanisms, offering deeper insights into how epigenetic remodeling shapes the regionalized immune response in CLDs like MASH and HCC. While scRNA-seq has greatly advanced the dissection of immune cell heterogeneity in CLDs, this technology relies heavily on tissue dissociation. Notably, fibrotic/cirrhotic liver samples are characterized by tissue stiffness and extensive extracellular matrix(ECM) deposition (30), which may introduce significant methodological biases. Additionally, immune cell dropout and insufficient capture efficiency of rare cell subsets could obscure the true features of the immune microenvironment, and these limitations require careful consideration in result interpretation.

### 3.2 Spatial distribution and disease regional specificity

#### 3.2.1 Portal tract region

**Figure 2:**
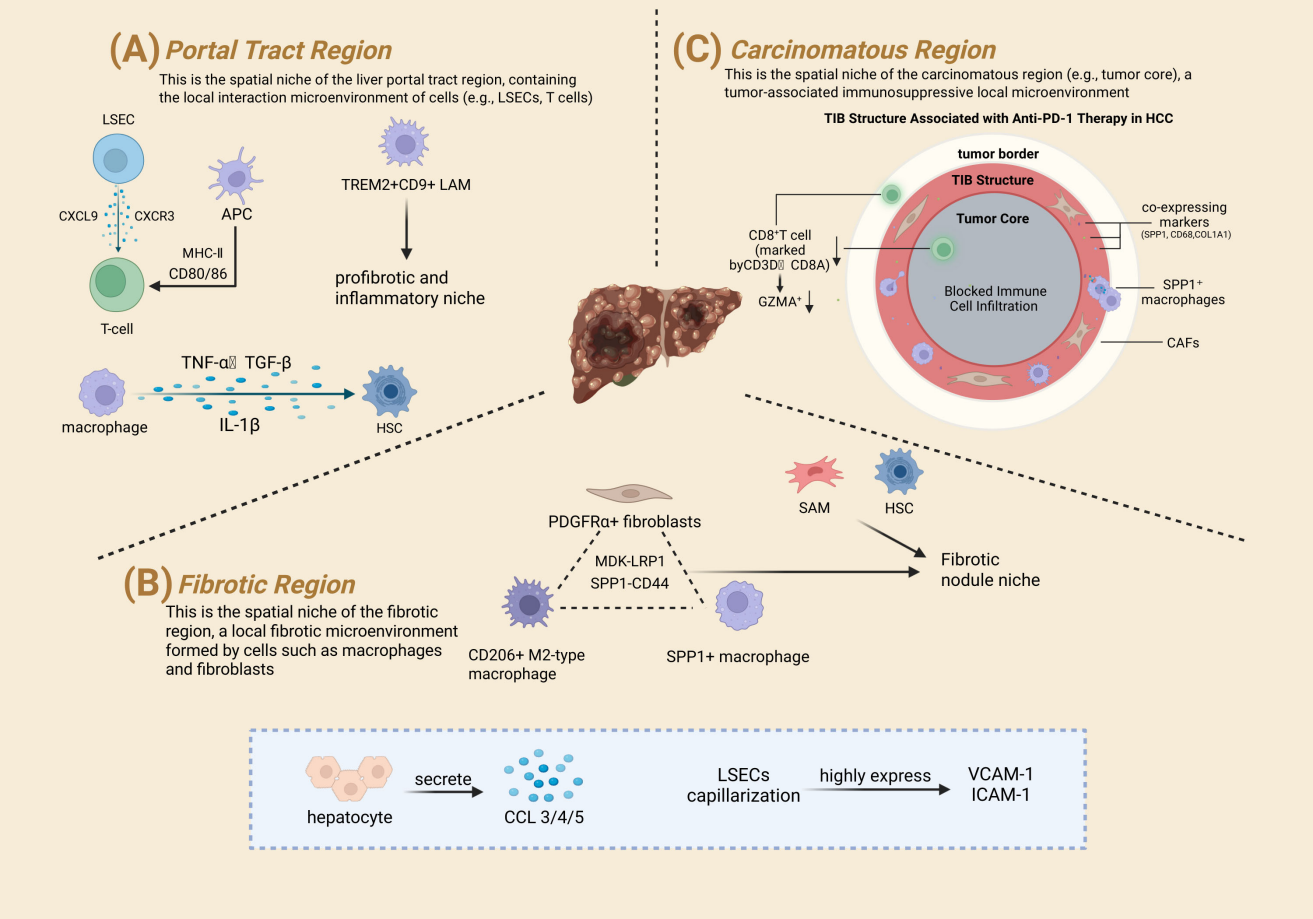
Immune microenvironment features of key regions in CLDs and shared vascular-immune regulatory mechanism. This Figure presents the immune microenvironment of three core anatomical regions in CLDs and their shared vascular-immune regulatory mechanism. **(A)** (Portal Tract Region): A key site for initiating immune responses. Inflamed LSECs secrete CXCL9 to recruit CXCR3^+^ T cells; activated macrophages release IL-1β, with upregulated TNF-α/TGF-β driving inflammation and HSC activation. APCs express high MHC-II and CD80/86 for T cell activation, and TREM2^+^CD9^+^ LAMs aggregate to form an inflammatory niche. **(B)** (Carcinomatous Region): Features a TIB at the tumor border—SPP1^+^ macrophages and CAFs (co-expressing SPP1/CD68/COL1A1) block infiltration of CD8^+^ T cells (marked by CD3D/CD8A/GZMA), mediating anti-PD-1 therapy resistance. **(C)** (Fibrotic Region): Has a “fibrotic niche”—PDGFRα^+^ fibroblasts, CD206^+^ M2 macrophages, and SPP1^+^ macrophages interact via MDK-LRP1/SPP1-CD44 to activate HSCs. 57 upregulated genes (e.g., TIMP1/COL1A1 for fibrogenesis) support its pro-fibrotic property.

In the portal tract region, inflammatory cytokines TNF-α and TGF-β expression is significantly upregulated, while activated macrophages release pro-inflammatory factors like IL-1β, synergistically driving local inflammation and stellate cell activation (31). Additionally, the chemokines CXCL9/CXCR3 are secreted by inflamed LSECs, promoting T lymphocyte recruitment to the liver (32). More importantly, antigen-presenting cells, upon activation, express high levels of MHC-II and costimulatory molecules CD80/CD86, which are essential for initial T cell activation and priming, indicating the portal tract is a key site for T cell initiation and immune response launch (31) (**Figure 2A**). Furthermore, spatial transcriptomic studies have revealed disease-specific niches within the portal tract. In fibrotic livers, this region is enriched with pro-fibrotic cells like ACTA2+ FABP4+ hepatic stellate cells(HSCs) and COL3A1+ scar-associated mesenchymal cells. Concurrently, immune cell subsets such as VCAM1+ macrophages, chemokine-secreting T cells, and activated B cells also specifically aggregate in the portal area. These cells collectively form a unique pro-fibrotic and inflammatory microenvironment. This discovery precisely anchors specific cell types to the anatomical location of the portal tract, providing key insights for understanding liver fibrosis mechanisms and developing targeted therapies (27). In MASH, a significantly expanded macrophage population marked by TREM2 and CD9, now recognized as lipid-associated macrophages (LAMs), specifically aggregates in the portal and fibrotic septal regions, forming a distinct LAM niche (33) (**Figure 2A**).

#### 3.2.2 Fibrotic regions

Regarding gene expression, studies using spatial transcriptomics have identified gene expression signatures specific to fibrotic regions. Fifty-seven significantly upregulated genes (p < 0.05) were detected in fibrotic areas, primarily involved in biological processes like fibrogenesis (TIMP1, COL1A1), immune function (HLA-DRA, CD74), antigen presentation (PSMB9, PSME2), and RNA translation (RPS27, RPL13). In terms of cellular spatial distribution, HSCs and scar-associated mesenchymal cells (SAMs) are significantly enriched in fibrotic regions. Specific monocyte subtypes like TM-1, TM-3, and Kupffer cells (KC-1) show a markedly increased proportion within fibrotic areas (27). Additionally, spatial transcriptomics has precisely characterized the “fibrotic niche” in human liver fibrosis samples. This niche is characterized by a tripartite interaction involving PDGFRα+ fibroblasts, CD206+ M2-type macrophages, and SPP1+ (osteopontin) macrophages colocalizing at the intersections of fibrous septa. These cells highly express pro-fibrotic L-R pairs, such as MDK-LRP1 and SPP1-CD44, forming a core signaling network that drives persistent fibrosis progression (7) (**Figure 2B**).

#### 3.2.3 Carcinomatous regions

In HCC, spatial transcriptomics reveals high intratumoral heterogeneity. Combined single-cell and spatial transcriptomic studies identified a specific spatial structure in the tumor immune microenvironment of non-responders to anti-PD-1 therapy: the tumor immune barrier (TIB). This structure is formed by the spatial co-localization of SPP1^+^ macrophages and cancer-associated fibroblasts (CAFs) at the tumor border, co-expressing macrophage markers (SPP1, CD68) and CAF markers (COL1A1). Spatial transcriptomic data show a significant lack of CD8^+^ T cells (marked by CD3D, CD8A) and their cytotoxic effector molecules (e.g., GZMA) within and surrounding this TIB structure, creating a physical barrier to immune cell infiltration. In contrast, responding HCC patients lack this distinct TIB structure, and immune cells infiltrate more readily into the tumor core (34). Thus, spatial transcriptomics directly identifies the TIB as a key spatial microenvironment structure at the HCC tumor edge associated with resistance to immunotherapy (**Figure 2C**).

### 3.3 Cellular interaction networks

#### 3.3.1 Immune cell-parenchymal cell interactions

In non-alcoholic steatohepatitis (MASH), lipid-laden hepatocytes recruit and activate macrophages by releasing inflammatory cytokines such as IL-1β and TNF-α, forming a positive feedback loop that exacerbates inflammation (35) (**Figure 2A**). Analysis of PSC liver tissue using 10x Visium spatial transcriptomics revealed transcriptomic signals for the biliary marker CK7 (KRT7) and the hepatocyte marker HNF4 specifically co-localized at the edges of fibrotic scars, forming a “scar-hepatocyte interface.” This interface coincides precisely with areas of dense immune cell infiltration, providing spatial evidence that transdifferentiating hepatocytes share the same microenvironment with infiltrating immune cells, a prerequisite for their interaction. In PSC fibrotic areas, hepatocytes are a significant source of key chemokines like CCL3, CCL4, CCL5, CXCL8, and CXCL16 (**Figure 2B**). The expression of these chemokines spatially correlates highly with the location of T cells and myeloid cells expressing corresponding receptors (e.g., CCR5, CXCR6). This provides direct spatial evidence that hepatocytes in PSC are not passive victims but actively secrete signaling molecules, creating a chemical gradient field that directly guides and recruits specific immune cell subsets to the lesion sites (28).

#### 3.3.2 Immune evasion mechanisms in the tumor microenvironment

In certain HCC subtypes, cancer cells highly express PD-L1 and form spatial clusters adjacent to CD8+ T cells, suggesting local immune checkpoint interactions. Spatial transcriptomic studies have shown that immune evasion in HCC is closely linked to the deep spatial architecture of its microenvironment. This technology visually demonstrates that functionally exhausted T cells and immunosuppressive cells are not randomly scattered but form spatially confined “immunosuppressive niches” within the tumor. Furthermore, spatial transcriptomes precisely map the territories of different tumor subclones, revealing spatially heterogeneous defects in antigen presentation mechanisms and PD-L1 expression, creating “blind spots” for immune recognition. Additionally, by mapping gene expression back to tissue location, studies found that metabolically stressed regions (e.g., hypoxic zones) spatially overlap with areas of T cell functional suppression. These findings collectively confirm that immune evasion is governed by a series of spatially organized local microenvironments rather than being a globally uniform state (36).

#### 3.3.3 Vascular microenvironment and immune regulation

Liver sinusoidal endothelial cells (LSECs) undergo capillarization in CLD, losing their characteristic fenestrations and highly expressing vascular adhesion molecules like VCAM-1 and ICAM-1, facilitating immune cell infiltration (**Figure 2B**). Spatial transcriptomic analysis further shows that LSECs and KC interact closely within the space of Disse, jointly regulating local immune responses (28). Spatial transcriptomics has revealed the spatial heterogeneity of sinusoidal capillarization. In MASH-related fibrosis, analysis of transcriptomic data captured from fibrotic septa regions found significant enrichment of a gene expression signature for capillarization markers, with CD34 being one core gene (7). Combined with spatial proteomics, research found that periportal stromal cells (particularly fibroblasts) highly express VCAM1 and CCL2. These cytokines and adhesion molecules co-localize spatially with recruited monocytes/macrophages (LAMs), constituting a microenvironment that recruits monocytes and promotes their retention (33). Spatial transcriptomics addresses the lack of spatial context inherent in single-cell technologies, but the current upper limit of spatial resolution, coupled with the fact that L-R interaction predictions are mostly based on gene expression correlations, renders them prone to over-interpretation. Furthermore, impaired tissue integrity in fibrotic samples may compromise the accuracy of gene localization. These issues have not been fully resolved in existing studies and could lead to misjudgment of immune-stromal cell interactions.

## 4 Organoids

### 4.1 Technical advantages

While single-cell and spatial transcriptomics have greatly deepened our understanding of the *in vivo* immune microenvironment, causal validation of mechanisms and facilitating clinical translation still require *in vitro* models that highly mimic the complex structure and function of the human liver. Organoid technology demonstrates its irreplaceable value in meeting this need. Organoid technology involves culturing human liver cells *in vitro* to simulate tissue structure and function. Derived from patient-specific stem cells or primary cells, organoids retain key liver structures, such as biliary networks and lobular polarity, as well as critical functions like the expression of liver-specific metabolic enzymes and bile secretion, thereby enabling more authentic modeling of the disease microenvironment. Furthermore, this technology avoids the species-specific functional differences between traditional mouse models and the human liver, facilitating direct study of human-specific immune mechanisms. Additionally, organoids can be cultured at scale, making them suitable for large-scale drug library screening and personalized drug testing, for instance, to evaluate the effects of different adjuvants on immune responses.

### 4.2 Disease modeling and pathological mechanism studies

#### 4.2.1 Recapitulating human liver pathophysiology with organoids

Organoids can recapitulate liver morphology, metabolic functions, and gene expression profiles, providing high-fidelity models for studying immune mechanisms in CLDs. Mouse models repopulated with human liver tissue, comprising human hepatocytes and non-parenchymal cells (NPCs) including human immune cells, endothelial cells, and stellate cells, have revealed species-specific interactions. For example, WNT2 secreted by sinusoidal endothelial cells controls cholesterol uptake and bile acid conjugation in hepatocytes via the receptor FZD5, successfully modeling human cholesterol metabolism regulation. In highly humanized mouse models, human NPCs increased the expression of a set of cholesterol pathway genes in hepatocytes, successfully mimicking the pathological processes of CLDs like liver fibrosis and non-alcoholic liver disease (37).

#### 4.2.2 Cirrhosis modeling

Hepatic stellate cell (HSC) activation is a central event in liver fibrosis. By simulating processes like HSC activation and collagen deposition, organoids have revealed the regulatory roles of immune cells like macrophages and T cells within the fibrotic microenvironment. Induced pluripotent stem cell (iPSC)-derived HSCs (iPSC-HSCs) closely resemble primary human HSCs at the transcriptional, cellular, and functional levels. When co-cultured with HepaRG hepatocytes in 3D spheroids, iPSC-HSCs maintain a quiescent phenotype; however, upon stimulation with wound-healing mediators and hepatocyte toxicity, they become activated, mount a fibrotic response, and secrete procollagen, mimicking the *in vivo* activation of HSCs upon liver injury. Activated HSCs from 2D culture can be encapsulated within 3D collagen gels to form spherical structures. Compared to 2D-cultured LX-2 cells, this 3D structure creates a stiffer environment and expresses higher levels of molecules associated with collagen deposition and fibrosis, such as TIMP1 and LOXL2, simulating the process of *in vivo* collagen deposition and fibrosis progression.5.2.3 Fatty Liver Models.

Research teams worldwide have made significant progress using specific organoid models. Pingitore et al. established a multicellular 3D organoid composed of HepG2 cells and the HSC cell line LX-2 carrying the MASLD-associated PNPLA3 I148M homozygous mutation. Upon exposure to free fatty acids, these organoids developed fat accumulation, mimicking MASLD pathology. Ouchi et al. utilized iPSCs to establish a liver organoid model encompassing various liver cell types, including KCs, revealing the key role of macrophages in liver inflammation. Treatment with free fatty acids successfully recapitulated features of steatohepatitis, including steatosis, inflammation, and fibrosis. Ramli et al. generated an organoid system containing hepatocytes and biliary epithelial cells from iPSCs. When exposed to free fatty acids, these organoids exhibited gene expression profiles similar to those observed in liver tissue from MASH patients. Furthermore, they showed structural alterations, such as worsened biliary networks and ductular reaction, resembling features seen in MASH. Researchers have also used human fetal hepatocyte organoids to model the initiation of MASLD, specifically steatosis, triggered by free fatty acid load, individual genetic variation, and monogenic lipid disorders. This study included screening candidate drugs to identify compounds effective against steatosis and conducted mechanistic assessments, revealing inhibition of *de novo* lipogenesis as a common molecular pathway (38). Using patient-derived organoids(PDOs), researchers can observe damage to hepatocytes caused by autoantibodies or aberrant immune responses. Treating liver organoids with ethanol or free fatty acids can partially induce responses in KCs and co-cultured T lymphocytes, including migration, cytokine release, and cell-cell interactions (38). Several hepatitis organoid models have now been developed to simulate disease phenotypes and screen therapeutic approaches. Recent advances in immune-engineered organoid systems—incorporating vascular or microfluidic components—have further enhanced the mimicry of the hepatic immune microenvironment. By integrating endothelial cells (to form vascular networks) and microfluidic channels (to simulate blood flow), these organoids recapitulate the vascular-immune crosstalk critical for immune cell infiltration and activation in CLDs. For example, vascularized liver organoids co-cultured with autologous T cells and macrophages have been used to model AIH, revealing how endothelial cell-derived VCAM-1 mediates T cell adhesion and infiltration into hepatocyte regions, leading to immune-mediated liver injury. Additionally, microfluidic-based immune-engineered organoids have enabled real-time monitoring of immune cell dynamics in response to pro-fibrotic stimuli, providing insights into the spatial regulation of immune-stromal interactions during liver fibrosis. These models address the limitations of conventional organoids and offer a more physiological platform for studying immune mechanisms and testing immunotherapies.

### 4.3 Drug screening and toxicity studies

#### 4.3.1 New drug testing: assessing efficacy of antioxidants or anti-inflammatory drugs in alcoholic liver disease models

Organoids show great promise not only in mechanistic research but also in drug development. By integrating HSCs and human fetal liver mesenchymal cells (hFLMCs) into human embryonic stem cell-derived hepatic organoids (hEHOs), researchers successfully modeled core pathological features of alcohol-associated liver disease (AALD) (39). The strength of this model lies in its stable expression of key alcohol-metabolizing enzymes ADH and CYP2E1, faithfully recapitulating the biochemical conversion of ethanol to acetaldehyde. More importantly, it triggers an oxidative stress cascade via ROS accumulation, inducing typical steatotic and fibrotic phenotypes, thus providing a precise pathological context for drug evaluation.

Ethanol exposure experiments validated the model’s dynamic response, which aligns well with clinical pathology: enhanced CYP2E1 activity (2.8-fold increase) accompanied by significantly elevated secretion of liver injury markers (ALT, AST, LDH; p<0.01), alongside a reduction in organoid viability to 43% of controls and the appearance of fibrotic features like collagen deposition. Subsequent drug mechanism studies using this model found that the antioxidant N-acetylcysteine (NAC) reduced liver injury marker levels by 56-72% (p<0.001) and restored organoid viability to 82% of normal levels by scavenging ROS. Further mechanistic analysis revealed that acetaldehyde, a key metabolic intermediate, activates KCs and HSCs, promoting the release of pro-inflammatory factors like IL-1β (reaching concentrations of 1.2-1.5 ng/mL). An IL-1 receptor antagonist, by blocking this signaling pathway, reduced the fibrotic area by 64% (p<0.005) (39). Overall, these findings not only validate the model’s sensitivity to antioxidant/anti-inflammatory drugs but also elucidate the molecular mechanisms by which ethanol metabolites drive liver injury through immune-metabolic crosstalk, providing a reliable experimental platform for developing novel therapeutic strategies.

#### 4.3.2 Toxicity prediction: more accurate prediction of drug-induced liver injury risk

Beyond efficacy evaluation, organoids also demonstrate significant advantages in drug safety assessment. In studies of drug metabolism function, organoid technology shows considerable application potential. Firstly, organoids express key metabolic enzymes, such as CYP450 family members and other phase II detoxification enzymes, at levels resembling liver tissue, enabling them to simulate drug metabolism processes. Studies utilizing acetaminophen, troglitazone, and trovafloxacin metabolism simulations have provided an experimental basis for investigating DILI mechanisms. Building on this, the organoid toxicity screening platform (LOT) developed by Shinozawa et al. further validated its predictive power: testing 238 marketed drugs, the platform demonstrated a sensitivity of 88.7% and a specificity of 88.9%, proving that organoids can accurately assess potential drug toxicity risks to the liver through their metabolic enzyme functions. Furthermore, dispersed human liver organoids (hLOs) based on iPSCs also possess drug metabolism capabilities, allowing quantitative determination of compound IC50 values, with DILI prediction performance comparable to intact organoids (40). This finding not only expands the application formats of organoids but also methodologically supports the central role of metabolic function simulation in predicting DILI risk. Complementing traditional organoid models, liver-on-chip microphysiological systems have emerged as a powerful tool for simulating hepatic physiology and pathology with enhanced clinical relevance. These microfluidic devices recapitulate key liver microenvironmental features—including hemodynamic flow, cell-cell crosstalk between hepatocytes, LSECs, and immune cells, and ECM stiffness—mimicking the *in vivo* spatial and mechanical cues critical for immune cell function. In CLD research, liver-on-chip models have been used to study MASH-related inflammation by co-culturing hepatocytes, HSCs, and macrophages in a microfluidic environment, revealing how shear stress regulates pro-inflammatory cytokine secretion and macrophage polarization (41). For drug screening, liver-on-chips have demonstrated superior predictive power for DILI compared to 2D cultures, as they replicate the metabolic-immune crosstalk that modulates drug toxicity (42).

### 4.4 Personalized medicine and immunotherapy exploration

#### 4.4.1 Immunomodulatory therapy: testing responsiveness to immunosuppressants

The high customizability of organoid models paves the way for their application in personalized medicine. Organoids can serve as platforms for studying interactions between immune cells and hepatocytes, suitable for screening therapeutic strategies for diseases like immune-mediated liver injury. Drug sensitivity testing on patient-derived PDOs can help determine individualized treatment regimens, strongly suggesting their utility for testing responsiveness to immunosuppressants. In summary, organoids can model immune dysregulation-mediated diseases like AIH, and the optimization of immunotherapies such as PD-1/CTLA-4 inhibitors can benefit from organoid models (43).

#### 4.4.2 Gene editing and cell therapy

Further, the combination of organoids with gene editing technologies provides a powerful tool for developing novel cell therapies. CRISPR technology enables precise gene editing in organoid cells, allowing modeling of gene mutations or correction of defects, thereby studying the impact of such edits on immune cell infiltration, cytokine secretion, and other aspects within the immune microenvironment. Simultaneously, optimizing the immune compatibility of organoids via gene editing can provide safer targeting models for cell therapies like CAR-T (44). Organoid models have been used to validate the killing efficacy and tumor specificity of CAR-T cells, indicating their potential as platforms for targeted validation of immune cell therapies. CRISPR can further engineer organoids to simulate specific gene mutations, such as tumor driver genes, and study their effects on T cell infiltration, immune checkpoint expression, etc., within the immune microenvironment, thereby optimizing CAR-T treatment strategies (43). Organoid models provide a high-fidelity platform for mechanistic validation and drug screening in CLDs, but they still exhibit significant interpretational gaps. On one hand, the lack of vascularization and a complete immune cell infiltration system makes it difficult to fully recapitulate the complex immune-metabolic crosstalk *in vivo*. On the other hand, the lack of standardization in culture conditions across different laboratories results in poor reproducibility of some research findings and even conflicting conclusions, which need to be standardized in future studies.

## 5 Limitations, controversies, and future directions

Despite the transformative advances brought by scRNA-seq, spatial transcriptomics, and organoid technology in CLD immunomicroenvironment research, the field remains constrained by significant methodological limitations, interpretational gaps, and conflicting findings. A dedicated critical examination of these issues is essential to avoid over-interpretation, balance the review’s descriptive focus, and guide future research toward greater rigor and reproducibility.

### 5.1 Methodological biases in tissue dissociation of fibrotic/cirrhotic samples

Tissue dissociation is an indispensable prerequisite for scRNA-seq but introduces inherent, disease-specific biases—particularly in fibrotic or cirrhotic liver tissues, the core research objects of CLD. Excessive ECM deposition and tissue stiffening in these samples severely hinder the penetration of dissociation enzymes such as collagenase and dispase, leading to two critical artifacts that distort the true immune microenvironment. Selective cell loss is a major issue: activated HSCs, KCs tightly adherent to sinusoidal endothelial cells, and dendritic cells (DCs) embedded in fibrotic septa are prone to incomplete dissociation, resulting in underrepresentation of these key immunoregulatory cells in scRNA-seq datasets and overestimation of easily dissociated cells like circulating T cells (45). Additionally, prolonged dissociation time induces stress responses in immune cells, upregulating genes related to inflammation and apoptosis, leading to misclassification of “stress-induced activated cells” as “disease-specific activated cells” and confounding interpretations of *in vivo* immune cell function. Comparative studies show that dissociation protocols optimized for normal liver tissue yield a lower recovery rate of tissue-resident macrophages in cirrhotic samples compared to fibrosis-tailored protocols, highlighting how the lack of disease-specific standards exacerbates result heterogeneity. Notably, neutrophils—critical for hepatic inflammation in alcoholic hepatitis and MASH—exhibit distinct recovery biases: their fragile cytoplasmic structure and rapid activation upon tissue manipulation cause significant lysis during dissociation, with recovery rates lower than in normal liver tissue (46). This uneven recovery not only underestimates their proportion in the intrahepatic immune microenvironment but also obscures functional heterogeneity, further distorting understanding of their role in CLD pathogenesis.

### 5.2 Immune cell dropout and underestimation of rare cell subsets

Immune cell dropout—where low-abundance transcripts or fragile immune cells fail to be captured by sequencing platforms—poses a major challenge for scRNA-seq-based CLD research, especially in identifying rare but functionally critical subsets. LAMs and DNT cells account for only 1–3% in the peripheral blood and lymphoid organs, and their detection relies heavily on high RNA capture efficiency (47). Studies using different scRNA-seq platforms (e.g., 10x Genomics vs. Smart-seq2) report conflicting frequencies of these subsets, with Smart-seq2 showing a higher DNT cell detection rate due to improved transcript coverage, suggesting platform-specific limitations may lead to underestimation or omission of key rare populations (48). Another issue is the misclassification of circulating vs. tissue-resident cells: peripheral T cells and other circulating immune cells are more prone to detachment during tissue processing, leading to their overrepresentation in scRNA-seq datasets. This bias is amplified in cirrhotic livers with increased vascular permeability, making it difficult to distinguish truly tissue-resident immune cells from transient circulating cells and misleading interpretations of “liver-specific immune responses.

### 5.3 Inherent limits of spatial resolution and tissue integrity

Spatial transcriptomics addresses the lack of spatial context in scRNA-seq but faces inherent technical constraints and disease-related tissue damage in CLD applications. The spatial resolution bottleneck remains unresolved: even high-resolution platforms (e.g., Visium HD, Xenium) have spot sizes encompassing 2–5 cells in dense regions like portal tracts, making it impossible to assign transcripts to individual cells or verify direct cell-cell contacts. For example, predicted interactions between macrophages and HSCs at the fibrotic front may merely reflect co-localization within the same spot rather than true functional crosstalk. RNA degradation is an underappreciated issue exacerbating biases: fibrotic and cirrhotic tissues require prolonged fixation and decrosslinking due to dense ECM, accelerating the degradation of short-lived, immune-regulatory transcripts. RNA degradation is an underappreciated issue exacerbating biases: fibrotic and cirrhotic tissues require prolonged fixation and decrosslinking due to dense ECM, accelerating the degradation of short-lived, immune-regulatory transcripts. This degradation pattern introduces unavoidable bias when quantifying immune cell activation states, which rely on accurate detection of short-lived cytokines and chemokines (spatial transcriptomics) (27). Tissue integrity compromise further limits utility: severe architectural distortion and ECM cross-linking in fibrotic/cirrhotic tissues make intact sectioning difficult, resulting in fragmented samples and lost spatial context in fibrotic septa, which directly impairs the accuracy of gene expression localization. Additionally, spatial transcriptomics cannot distinguish between membrane-bound and soluble ligands, leading to ambiguous interpretation of paracrine vs. autocrine signaling—for instance, predicted PD-L1/PD-1 interactions in HCC may reflect soluble PD-L1 secretion rather than direct cell-cell binding.

### 5.4 Over-interpretation of L-R predictions and conflicting results

L-R interaction prediction is a core tool for inferring intercellular communication but is frequently over-interpreted in CLD research, leading to misleading conclusions. Most studies rely on computational tools such as CellPhoneDB and NATMI, which use gene co-expression as the sole criterion without validating protein expression, binding affinity, or functional relevance. For example, the predicted MDK-LRP1 interaction between fibroblasts and macrophages in fibrotic niches has not been confirmed by co-immunoprecipitation or neutralization experiments, leaving open the possibility of false-positive predictions. Conflicting results across studies further highlight the need for critical reflection: regarding Tregs in PBC, some studies report Treg dysfunction, while others suggest Tregs retain immunosuppressive capacity but are sequestered by stromal cells—a discrepancy likely driven by differences in tissue dissociation protocols and L-R prediction thresholds (49, 50). Similarly, the pro-fibrotic role of SAMs is well-established in human cirrhosis (51), but murine models show inconsistent results (52), with some reporting anti-fibrotic SAM subsets, highlighting species-specific limitations and the need for cross-validation. Notably, most predicted interactions lack functional validation via targeted approaches such as neutralizing antibody blocking, CRISPR-mediated gene knockout, or *in vitro* co-culture assays. This gap between computational inference and experimental confirmation risks misidentifying non-physiological interactions as therapeutic targets, hindering clinical translation.

### 5.5 Limitations of organoid models in recapitulating hepatic physiology

Current liver organoid systems, despite their value in disease modeling, fail to replicate key physiological features of the human liver, severely restricting their translational relevance. Vascularization is absent in most conventional organoids, precluding the simulation of immune cell recruitment via the circulatory system, ischemia-reperfusion injury, and vascular-immune crosstalk that drives fibrosis progression. Bile-flow physiology is also not recapitulated, as organoids lack intact biliary excretion pathways—limiting the modeling of cholestatic liver diseases where bile stasis-induced immune activation is a core pathogenesis. Immune-cell longevity is compromised: primary immune cells co-cultured with organoids survive shortly, making it impossible to study long-term immune responses such as chronic exhaustion or immune memory formation. Additionally, metabolic zonation—the regional specialization of hepatocytes—is absent, failing to replicate the spatial heterogeneity of metabolic-immune crosstalk that regulates liver inflammation and fibrosis. These physiological limitations mean organoid-based findings may not accurately reflect *in vivo* immune microenvironment dynamics.

### 5.6 Computational tools and interpretational gaps in multi-omics integration

Multi-omics integration is hailed as a solution to unravel complex immune regulatory networks but introduces significant interpretational gaps in CLD research. Discrepancies between omics layers are common: scRNA-seq may detect upregulation of pro-inflammatory genes in macrophages (53), but epigenomic data may show reduced chromatin accessibility at these loci—indicating transcriptional priming rather than active expression—yet most studies fail to reconcile such discrepancies, leading to oversimplified conclusions about cell function. Integrating multi-omics datasets is also computationally intensive due to the high dimensionality of single-cell data and the need for complex algorithms for batch correction, cell matching, and interaction inference, which requires advanced computational resources and specialized expertise not accessible to most research teams. The lack of standardized computational tools further exacerbates the issue: different studies use distinct algorithms for batch correction and integration, leading to variable cell clustering outcomes and hindering cross-study comparisons.

Core computational tools for CLD multi-omics analysis include functional modules tailored to different data types: single-cell analysis frameworks for scRNA-seq preprocessing, dimensionality reduction, and cell-type annotation, with Harmony and scVI critical for batch correction; spatial analysis suites for spatial clustering, cell-type deconvolution, and immune-stromal interaction mapping; L-R inference tools for constructing communication networks; and trajectory/lineage tools for modeling cell state transitions. However, key challenges persist: batch correction and donor variability confound datasets, sparse sampling from scarred tissue compromises trajectory inference and L-R analysis, inconsistent cell-type annotation stems from the lack of a unified liver-specific immune reference atlas, and bridging *in vivo* and *in vitro* data remains hindered by experimental system differences.

### 5.7 Future directions to address current challenges

Building on the critical reflection of these limitations, targeted future directions are needed to advance CLD immunomicroenvironment research toward precision and reproducibility. Technological innovations should focus on developing high-sensitivity, low-damage single-cell capture technologies to address small biopsy sizes and challenges in isolating intrahepatic immune cells. For spatial transcriptomics, optimizing tissue processing protocols for fibrotic/cirrhotic samples—including improved fixation and RNA protection methods—will enhance gene localization accuracy. Organoid model optimization should prioritize functional enhancement and standardization of co-culture conditions to improve reproducibility.

Multi-technology integration strategies will be key: combining scRNA-seq and spatial transcriptomics to construct 3D maps of “cell type - spatial location - functional state,” and integrating metabolomics with immunology to reveal how metabolic reprogramming regulates the immune microenvironment. Computationally, developing donor-aware integration tools that account for clinical covariates, CLD-tailored sparse data imputation methods, and a unified liver-specific immune reference atlas will standardize analyses and enable cross-study comparisons. Finally, strengthening the link between computational predictions and experimental validation—via functional assays of key L-R interactions and cell trajectories—will ensure that discoveries translate reliably to clinical applications, advancing personalized immunotherapy for CLD.

## 6 Conclusion

In summary, the synergistic application of scRNA-seq, spatial transcriptomics, novel immunotherapeutics, and organoid technologies provides a comprehensive pipeline for immunological research in CLDs, spanning from mechanistic dissection to personalized therapy validation. Looking ahead, further optimization of technical details, combined with the subtype characteristics of CLDs like cirrhosis, fatty liver disease, alcoholic liver disease, and autoimmune liver disease, is needed to propel immunological research towards precision and personalization.

## Glossary

AALD: Alcohol-Associated Liver Disease
ADH: Alcohol Dehydrogenase
AIH: Autoimmune Hepatitis
ALT: Alanine Aminotransferase
AMA: Anti-Mitochondrial Antibodies
ANA: Antinuclear Antibodies
APCs: Antigen-Presenting Cells
AST: Aspartate Aminotransferase
BAFF: B-cell Activating Factor
BCR: B Cell Receptor
CAFs: Cancer-Associated Fibroblasts
CAR-T: Chimeric Antigen Receptor T-Cell
CHB: Chronic Hepatitis B
CHC: Chronic Hepatitis C
CLD: Chronic Liver Disease
CRISPR: Clustered Regularly Interspaced Short Palindromic Repeats
CTLA-4: Cytotoxic T-Lymphocyte-Associated Protein 4
CXCL: Chemokine (C-X-C Motif) Ligand
CYP2E1: Cytochrome P450 2E1
DBiT-seq: Deterministic Barcoding in Tissue for sequencing
DCs: Dendritic Cells
DILI: Drug-Induced Liver Injury
DNT: Double-Negative T (cells)
ECM: Extracellular Matrix
hEHOs: Human Embryonic stem cell-derived Hepatic Organoids
hFLMCs: Human Fetal Liver Mesenchymal Cells
HCC: Hepatocellular Carcinoma
HCV: Hepatitis C Virus
HNF4: Hepatocyte Nuclear Factor 4
HSCs: Hepatic Stellate Cells
ICAM: Intercellular Adhesion Molecule
IL: Interleukin
iPSC: Induced Pluripotent Stem Cell
iPSC-HSCs: Induced Pluripotent Stem Cell-derived Hepatic Stellate Cells
ISG: Interferon-Stimulated Gene
KCs: Kupffer Cells
KRT7: Keratin 7 (a biliary marker, CK7)
LAG3: Lymphocyte-Activation Gene 3
LAMs: Lipid-Associated Macrophages
LDH: Lactate Dehydrogenase
LKM1: Liver-Kidney Microsomal type 1
LOT: Liver Organoid Toxicity (screening platform)
LOXL2: Lysyl Oxidase Homolog 2
LPAMs: Liver Portal-Associated Macrophages
LSECs: Liver Sinusoidal Endothelial Cells
L-R: Ligand-Receptor
MASLD: Metabolic Dysfunction-Associated Steatotic Liver Disease
MASH: Metabolic Dysfunction-Associated Steatohepatitis
MDK: Midkine
MHC: Major Histocompatibility Complex
NAC: N-Acetylcysteine
NETs: Neutrophil Extracellular Traps
NKT: Natural Killer T Cells
NPCs: Non-Parenchymal Cells
PBC: Primary Biliary Cholangitis
PDGF: Platelet-Derived Growth Factor
PD-L1: Programmed Death-Ligand 1
PDOs: Patient-Derived Organoids
PGE2: Prostaglandin E2
PNPLA3: Patatin-like phospholipase domain-containing protein 3
PSC: Primary Sclerosing Cholangitis
ROS: Reactive Oxygen Species
SAMs: Scar-Associated Macrophages
TACI: Transmembrane Activator and CAML Interactor
TAMs: Tumor-Associated Macrophages
TCR: T Cell Receptor
Tfh: T Follicular Helper
TGF: Transforming Growth Factor
TIB: Tumor Immune Barrier
TIM: T-cell Immunoglobulin and Mucin Domain
TIMP1: Tissue Inhibitor of Metalloproteinase 1
TLS: Tertiary Lymphoid Structures
TNF: Tumor Necrosis Factor
Tregs: Regulatory T Cells
VCAM: Vascular Cell Adhesion Molecule
YAP/TAZ/CYR61: Yes-associated Protein/Transcriptional Coactivator with PDZ-binding motif/Cysteine-Rich Angiogenic Inducer 61.
